# Human-attacks by an urban raptor are tied to human subsidies and religious practices

**DOI:** 10.1038/s41598-019-38662-z

**Published:** 2019-02-22

**Authors:** Nishant Kumar, Yadvendradev V. Jhala, Qamar Qureshi, Andrew G. Gosler, Fabrizio Sergio

**Affiliations:** 1 Edward Grey Institute of Field Ornithology, Department of Zoology; South Parks Road, University of Oxford, Oxford, OX1 3PS United Kingdom; 2 Wildlife Institute of India, Post Box # 18, PIN- 248001 Chandrabani, Dehradun, (Uttarakhand) India; 3 Institute of Human Sciences, School of Anthropology and Museum Ethnography, 58a Banbury Rd., Oxford, OX2 6QS United Kingdom; 4 Department of Conservation Biology, Estacion Biologica de Doñana-CSIC, C/ Americo Vespucio 26, 41092 Sevilla, Spain

## Abstract

Growing urbanization is increasing human-wildlife interactions, including attacks towards humans by vertebrate predators, an aspect that has received extremely scarce investigation. Here, we examined the ecological, landscape and human factors that may promote human-aggression by raptorial Black kites *Milvus migrans* in the 16-millions inhabitants megacity of Delhi (India). Physical attacks depended on human activities such as unhygienic waste management, ritual-feeding of kites (mainly operated by Muslims), human density, and presence of a balcony near the nest, suggesting an association between aggression and frequent-close exposure to humans and derived food-rewards. Surprisingly, while more than 100,000 people could be at risk of attack in any given moment, attitudes by local inhabitants were strikingly sympathetic towards the birds, even by injured persons, likely as a result of religious empathy. These results highlight the importance of socio-cultural factors for urban biota and how these may radically differentiate the under-studied cities of developing countries from those of western nations, thus broadening our picture of human-wildlife interactions in urban environments. The rapid sprawling of urban and suburban areas with their associated food-subsidies is likely to increase proximity and exposure of large predators to humans, and vice versa, leading to heightened worldwide conflicts.

## Introduction

There is growing interest in the interactions between human culture and animals, as evidenced by the rapid spread of studies in the field of ethnozoology^1–3^. The need for integration of human socioeconomic and cultural variables into ecological research is particularly obvious in studies that focus directly on human-wildlife conflicts, or on expanding anthropogenic environments such as cities, where urban residents are confronted with a “novel” human-wildlife interface^4–7^.

In particular, worldwide urban residents are experiencing a growing frequency of encounters with wildlife due to increasing urbanization, human encroachment of natural habitats, expanding greenspaces within cities, intentional feeding to attract wildlife, and growing adaptation of animal species to urban ecosystems^8–10^. While close encounters may be beneficial in reconnecting urban people with ‘nature’^11,12^, such increasing contacts are accompanied by an equally growing rate of human-wildlife conflicts, such as vehicle collisions, property damage, pet predation, disease transmission and even physical attacks on humans^13–15^. Conflicts of this kind are typically difficult to manage because socio-political and cultural attitudes and perceptions often make mitigation controversial^5,16^. This is especially pronounced in urban settings, which may pool together people with very different cultural backgrounds and with substantial differences in their interest or tolerance of wildlife, let alone of nuisance animals^14,17,18^. Furthermore, urban animals may behave differently from their rural counterparts, thus requiring specially-designed mitigation measures^19^.

An extreme and sometimes dramatic form of human-wildlife conflict is represented by direct physical attacks on humans, which may cause psychological distress, diseases, injuries, sometimes severe or permanent ones, and even loss of life^7,20–23^. Similarly to other forms of conflict, the frequency and severity of aggression on humans seem to be increasing in many urban areas^24–27^. This creates an urgent need to know the potential drivers and risk factors underpinning the attacks, in order to devise mitigation strategies and avoid conflict, which might prejudice peoples’ perceptions of and actions towards wildlife in general^28–30^.

**Figure 1:**
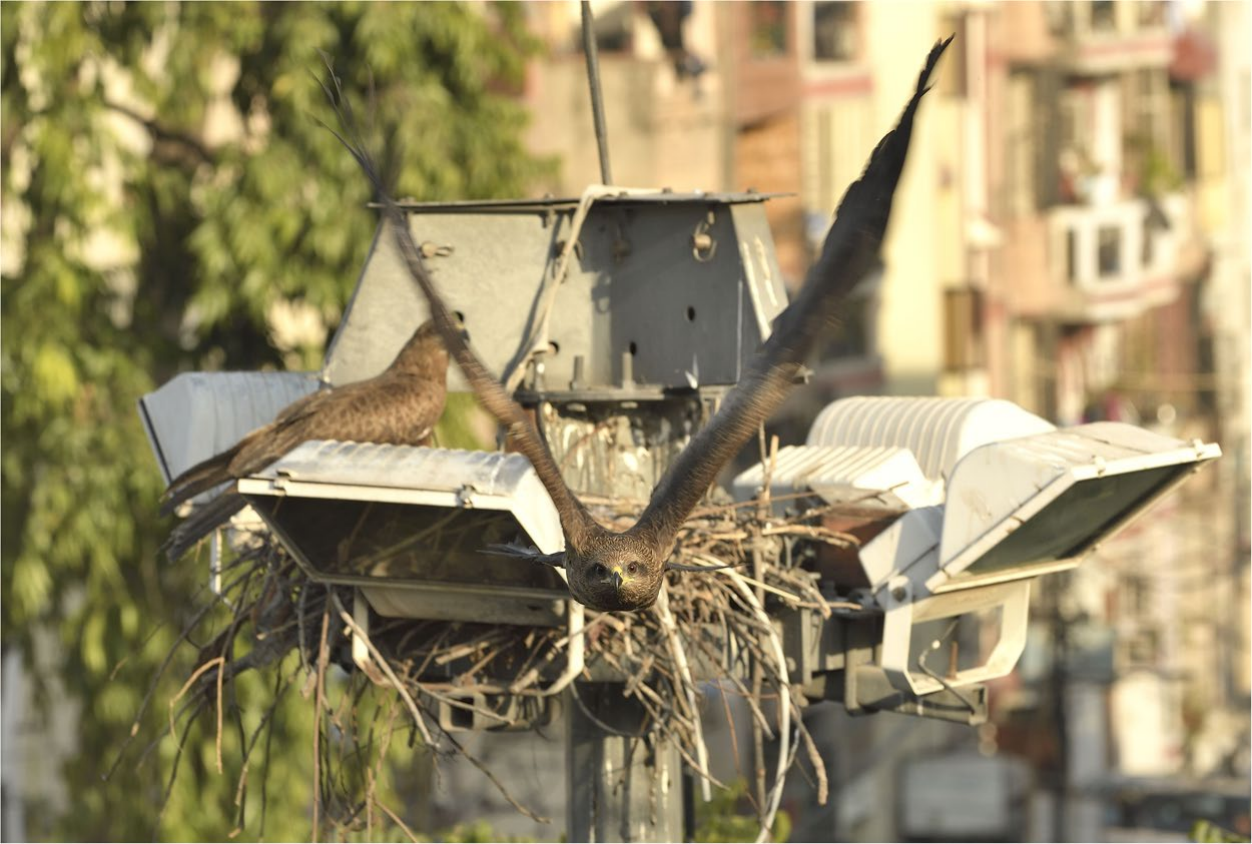
A Black kite takes off from its nest on a light pole to attack the photographer, who is standing on a balcony (Photo credit: F. Sergio).

A special subset of these potentially-aggressive species is represented by vertebrate top predators, such as mammalian carnivores or birds of prey. Because of their armament, harm potential, and dangerous iconic nature in collective imagery, these species typically evoke more emotional responses and intolerance than other species^31–33^, frequently leading to “hyper-perception of risk”^5^. For example, Kellert^34^ showed that people tend to be more afraid of species that have the potential to harm them. Independently of perceptions, some of these predators have colonized many cities and increased dramatically in some urban areas in recent decades, sometimes reaching densities unparalleled in rural areas^24,32,35–37^. In turn, this has increased encounters and conflict, sometimes with well demonstrated upturns in attack rates and even fatalities, frequently sensationalized by the public media^24–27^. In many cases, increased aggression has been linked with intentional and unintentional human feeding of the predators, resulting in consequent loss of fear^38–40^. Despite all the above, very few studies have examined the conditions that may predispose certain individuals to attack humans, and most of them have focused on mammals in rural areas^7,41–43^. Thus, there is a great need for information from urban areas and other taxonomic groups to advance knowledge in this field. Here we assess the ecological, landscape and human factors that may promote aggression towards humans by raptorial Black Kites *Milvus migrans* (Fig. 1) in Delhi (India), a megacity of 16-million inhabitants, which hosts one of the largest concentrations of vertebrate predators of the world^44^. In particular, we tested the hypothesis that individuals more exposed to human feeding and subsidies may be more likely to attack humans.

The Black Kite (hereafter “kite”) is a medium-sized, opportunistic predator and facultative scavenger. It is considered the most successful raptor in the world, due to its capability to withstand anthropogenic habitat change and even breed in high numbers alongside dense human populations within cities, especially in tropical areas^45^. Throughout its distribution, there are reports of individuals snatching food from humans, sometimes in aggressive ways, up to the point of being considered a local nuisance^46,47^. In India, the native, resident subspecies *M. m. govinda* is synurbic^48^, i.e. it occurs almost exclusively in close association with humans in towns and cities^49^. In Delhi, where this study was conducted, kites breed throughout the city, often a few meters from human habitation, and locally reach extremely high densities, thanks to the exploitation of human food subsidies facilitated by inefficient refuse disposal and by religious kite-feeding practices^44,50^. These centuries-old religious offerings (hereafter termed ‘ritualized-feeding’) consist in throwing meat scraps into the air for the kites to catch and are made for a variety of reasons, such as asking for blessings and relief from sins and worries^51,52^. Whilst meat-offering is practiced by a number of communities, in Delhi it is especially prevalent amongst members of Islamic faith, whose numbers are concentrated in well-defined portions of the city (hereafter ‘Muslim colonies’) where large quantities of meat are tossed to kites at predictable hours each day, sometimes causing hundreds of kites to congregate. Breeding individuals of this kite population often dive-bomb, scratch and harm humans with their talons when these approach their nest, sometimes causing deep cuts (Fig. 1). At times, these injuries may require medical examination because of the potential of subsequent infections, given kites’ frequent foraging on rotting organic waste.

## Results

To investigate the determinants of kites’ attacks on humans, we recorded aggressive events during routine visits to kites breeding sites, in which nests were approached and examined by a team of three people in a standardized manner (see Methods). Kites were classified as attacking when they dive-bombed and made physical contact with any member of the research team. In the four years of research, the percentage of attacking pairs averaged 25.5% (range 18.0–37.7%), and attacking individuals were present at 36 (i.e. 31.9%) of 113 separate territories checked at least once for reproduction. Twenty-one of these 36 attacking pairs but none of the 36 non-attacking pairs had a history of past local attacks, as from interviews with local inhabitants (χ^2^ = 15.09, P < 0.0001), suggesting that pairs identified as aggressive by our trials were not responding to an unusual stimulus, but were already known to be problematic pairs well before our research activities. In all attacks during our trials, kites dive-bombed at high speed and tried to hit the target-person on the head, typically with the open hallux and closed phalanges, so as to either scratch or knock the target-person on the head. Due to escape movements, scratches were sometimes re-directed on the forehead or on the neck or shoulders. All attacks were from behind and never when a person was staring at a kite dive-bombing towards the group.

**Figure 2:**
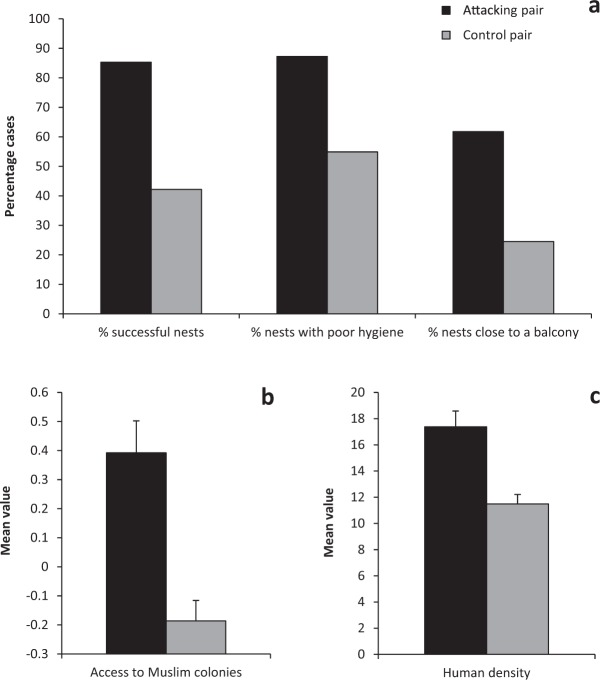
The likelihood of attacking humans by a breeding Black kite pair increased with: its breeding success (panel a, left bars), with more human waste around its nest (higher hygiene score, panel a, central bars), with the presence of a balcony in close proximity of the pair’s breeding site (panel a, right bars), with higher access to ritual subsidies from Muslim colonies (panel b), and with higher human density in the streets of the nest surroundings (panel c). Error bars represent 1 SE.

**Figure 3:**
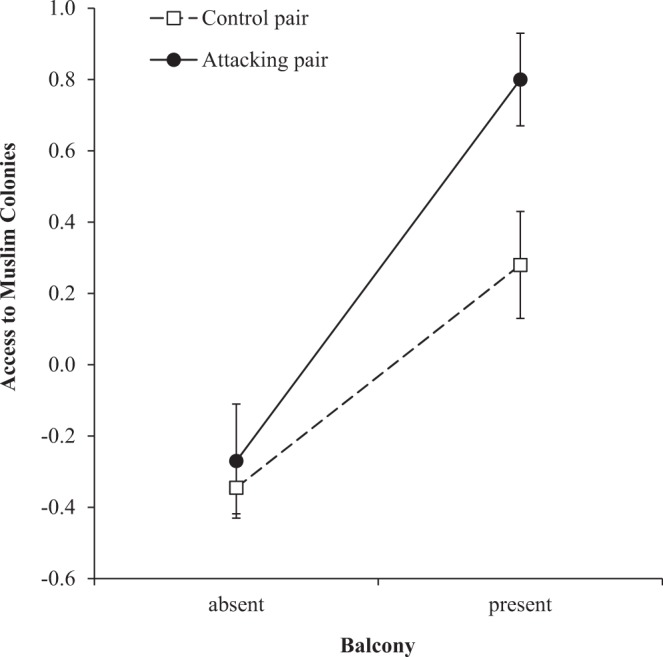
Likelihood of aggressive attack on humans by nesting Black kites in relation to access to ritual-feeding sites (Muslim colonies) and the presence of a balcony within 20 m radius of the nest. Error bars represent 1 SE.

To investigate the potential drivers of aggression, we built a logistic mixed model discriminating between attacking and non-attacking kites on the basis of a series of ecological, landscape and human socio-religious variables (see Methods). In this model, the likelihood of attack increased with kite breeding success, with more human waste around the nest (higher hygiene score), with higher access to Muslim subsidies, with higher human density in the streets and with the presence of a close-by balcony facing the nest (Tables 1 and 2, Fig. 2). The interaction between balcony presence and access to Muslim subsidies was also important: kite aggression was more likely for pairs that had both a balcony close by and high access to Muslim subsidies (Fig. 3).

**Table 1:** Top ranking (i.e. with ΔAICc < 3) generalised linear mixed models with binomial errors and a logit link function testing the effect of environmental, urban and human variables on likelihood of aggressive attack against humans (attacking vs control pair) by a nesting kite pair (N = 204 trials from 72 independent territories).

Explanatory variables in each model^a^	Degrees of freedom	AICc	Delta AICc	Model weight
Hygiene score + Breeding success	5	66.29	0.00	0.34
Balcony + Hygiene score + Breeding success + Access to Muslim colonies + Balcony * Access to Muslim colonies	8	67.77	1.48	0.16
Hygiene score + Breeding success + Access to Muslim colonies	6	68.08	1.79	0.14
Balcony + Hygiene score + Breeding success	6	68.18	1.89	0.13
Hygiene score + Urban cover + Breeding success	6	68.41	2.12	0.12
Human density + Hygiene score + Breeding success	6	68.48	2.19	0.11

Territory-identity nested within plot-identity was fitted as a random effect to all models. See Supplementary Table S.1 for the description of explanatory variables.

^a^Variables presented to the model: Number of people, Balcony, Urban cover, Green cover, Hygiene score, Human density, Access to Muslim colonies, Access to Muslim colonies * Hygiene score, Access to Muslim colonies * Urban cover, Access to Muslim colonies * Green cover, and Access to Muslim colonies * Balcony.

**Table 2:** Model averaged coefficients for the explanatory variables that entered the top-ranking models of Table 1.

Variable	B ± SE	Z test	*P* value
Breeding Success	28.69 ± 7.33	3.91	<0.0001
Hygiene score	30.4 ± 7.9	3.86	0.0001
Access to Muslim subsidies	1.31 ± 4.1	0.32	0.75
Balcony	8.57 ± 7.77	1.1	0.27
Balcony * Access to Muslim colonies	17.19 ± 6.86	2.54	0.012
Human density	1.14 ± 0.19	5.94	<0.0001
Urban Cover	0.57 ± 7.46	0.08	0.94
Intercept	−76.2 ± 21.37	3.56	0.0003

## Discussion

Our results contribute to advance and integrate different fields of research such as ethnozoology, urban ecology and the resolution of human-wildlife conflicts. Below, we (1) explain the mechanisms that may generate the observed patterns and (2) discuss the importance of our findings for each of the above three disciplines.

Kite attacks on humans were not randomly distributed through the city and responded to a series of indicators of human activities, such as unhygienic management of waste disposal, Muslim ritual-feeding, and the intensity of human activity in the streets. This configuration of socio-religious features is preferred by the kites of Delhi because of its high food availability in the form of ritual subsidies and organic garbage^50^ and may have promoted aggression in three non-exclusive ways. First, kites feeding on ritual subsidies or organic waste (frequently accomplished side by side with indigent people digging for useful materials) are frequently in close proximity to people, which may have lowered their fear of humans. Second, close proximity was frequently rewarded with food, which may have reinforced such loss of fear. Third, aggression varied with the interaction between access to Muslim ritual-subsidies and the presence of a balcony in the immediate proximity of the nest (Fig. 3). Thus, Muslim subsidies increased aggression-likelihood more markedly for pairs that nested in the immediate proximity of a balcony and, conversely, the presence of a balcony heightened aggression for pairs with ready access to Muslim subsidies. This suggests that peak aggression was promoted by the synergy of these two exposures to frequent and close encounters with humans.

In addition, human attacks were linked to successful reproduction. This could be promoted by two non-exclusive mechanisms: (1) parents could have a sense of the quality of their parental investment (e.g. based on their own or their offspring body condition) and defend more fiercely when success-prospects are high, as shown in other species^53–55^; and (2) aggressive behaviour towards humans paralleled the capability to repel other more common nest predators, such as crows or monkeys^44^, leading to a lower probability of nest failure from predation. Independently of motivational or causational mechanisms, if human-attacking pairs are more productive, there is a possibility that such behaviour could become more frequent in the population in the future, particularly so if aggression propensity were genetically inherited or culturally transmitted (e.g. by young kites emulating their parents’ defense tactics once adult).

### Implications for urban ecology

Implications for urban ecology were clear and profound. First, kite aggression did not respond to landscape composition or other classical ecological variables, but rather to a series of socio-religious and cultural features. This represents a clear-cut example of the importance of integrating human cultural factors into research programs in urban ecology. While human presence and action is one of the most defining characteristics of urban ecosystems^4^, few studies on urban animals explicitly incorporate human culture and perceptions into their design^56,57^. When such aspects are tested, they are usually found to be key factors for urban ecology and conservation. For example, human socio-economic status has been shown to affect avian diversity, occurrence and distribution^58–60^, while human perceptions of affinity/aversion towards certain animals varied across an urban-rural gradient, with important repercussions for potential conservation action^61^.

Second, some studies have shown that bolder, more aggressive individuals are more likely to colonize urban environments^62–65^. These links have usually been shown in comparisons of urban vs rural populations. If we consider the propensity to attack humans observed in this study as a measure of boldness, then our data extend this urban-rural comparison to individual variation within a city. Under this scenario, boldness in human tolerance may continue to be a key modulator of urban adaptation and exploitation even long after the initial colonization of the urban environment.

Third, the occurrence of the attacks in a highly-subsidized and thus high-density animal population coexisting with a dense human population generated a problem of unusual magnitude. First of all, only 25–30% of kite pairs attacked humans, which compares with 19 and 73% of Australian magpies *Gymnorhina tibicen* and Masked lapwings *Vanellus miles*, two species also renowned for their attacks on humans in urban settings^20,66^. However, even if only one in three or four pairs attacked humans, the local high density of kites over a very large area (average of 15 pairs/km^2^)^44^ implies that Delhi could easily hold over 5600 aggressive pairs. If we further consider that human density is high in Delhi and that attacking pairs were disproportionately concentrated in areas of higher human density, several thousand people could be potentially exposed to kites’ attacks every year. For example, during our tests of approximately 20 min duration, there were on average 18 people in the immediate proximity of the nest of an attacking pair. If this figure is representative, then multiplying it by 5600 aggressive pairs would imply that more than 101,000 people could be passing/standing within attacking-radius of an aggressive kite pair basically in every given moment of the day. Furthermore, most of the pairs that attacked us had a clear history of past attacks on local inhabitants, implying that our type of deliberate nest intrusion did not somehow exaggerate the extreme attacks that we recorded. Attacking individuals were ‘problematic’ already well before our activities. Conflicts of this magnitude and concentration would be unlikely in any rural setting and underline how urban ecosystems may pose novel challenges and require new approaches to wildlife management and conservation^19^.

### Implications for research on human-wildlife conflict

This study confirmed and extended current knowledge on the drivers of human-attacks by vertebrate animals. First, habituation to human proximity and animal feeding have been frequently reported as drivers of aggression on humans by mammalian carnivores and primates^38–40,42,67^. Our findings support these views, extend them to avian predators and thus suggest that they may represent generalized drivers of potential aggression across distantly related taxa. Second, human conflict with predators has often been associated with food scarcity driving low-quality, food-deprived individuals in close contact with humans^7,66,68–71^. In our case, attacks were concentrated in optimal, preferred habitat, and perpetrated by more productive, likely higher-quality individuals. Such dynamics may be more typical of synanthropic urban predators, whose high-quality individuals may be drawn to an abundant food supply but get habituated to humans in the process of accessing it. While food availability in both cases may mediate aggression, its enactment by individuals of different quality and breeding-potential may have strong repercussions for future trends in aggression rates, with obvious forecasting and management implications. This confirms the importance of resource-distribution in wildlife-human conflicts^10,72^ and further remarks how the management of synanthropic or urban wildlife may require specially-designed techniques^19^.

Finally, social factors and cultural perceptions have been identified as important drivers of the intensity of human-wildlife conflict, but are seldom taken into consideration^5,7,42,61,73,74^. Our results not only stressed the importance of socio-religious variables as key drivers of the conflict, but also show how they can enter the equally key human-part of conflict resolution. In fact, despite the above-reported magnitude of the problem, interviews with 140 persons encountered under the nests of attacking pairs uncovered extremely positive attitudes of local inhabitants towards the birds, even by individuals who were previously injured (authors’ unpubl. data). Overall, most people expressed fear for the attacks, as logically expected, but 100% of the respondents justified and showed explicit sympathy for the kites. Sympathy was motivated in two ways: (1) kites were protecting their offspring (i.e. doing their duty of good parents), and (2) humans have destroyed and degraded natural habitats and wildlife has no option but to live with humans, implying the ultimate fault was of people rather than kites. In turn, local communities, all of them of either the Islamic or the Hindu faith, tied such empathy to religious views about kites and about wildlife in general. Muslims mainly revered kites as sort of sacred, given their role of “winged emissaries” that metaphorically take away towards the sky their sins, worries, or prayers, symbolized by the meat offered during ritual-feedings^51,52^. Hindus believe that a soul undergoes body transformations, that all life forms are thus connected to one ultimate god form and thus they respected kites as part of their wider tolerance to all wildlife species as god’s beings. Finally, positive attitudes were probably further promoted by the fact that (1) attacks only occurred during a minor, predictable part of the year (duration of about two months), (2) that most injuries were generally light due to local people learning to avoid certain sites, and (3) that people were usually well aware of the useful ecosystem service provided to their neighbourhood by kites, which in Delhi remove more than 3900 tons of organic waste per year (authors’ unpubl. data).

Whatever the underlying motivation, these positive attitudes clearly translated into actions. All people reported taking (non-harmful) action to avoid confrontations, which may further reinforce kite aggression through additional reward. This included avoiding the nest proximity, dissuading children from using the parks or certain sections of the park, or refraining from using the balcony until the nestlings fledged. Some schools and canteens have changed their rules to make children and customers eat their lunch inside the premises rather than outdoor close to an attacking pair. In one case, the husband of a woman who received a serious scratch on her face enclosed the balcony with a volleyball net in order to get protection while continuing to use the balcony. In all these cases, no attempts at retaliatory measures, such as nest removal or killing the birds, were ever noticed or reported. On few occasions, local inhabitants enquired the possibility that we could remove the nest, but strongly specifying that it would have to be done after the nestlings had fledged. They also asked information about whether there could be non-harmful ways to dissuade kites from nesting at specific locations and about how to behave so as to avoid being attacked. Note that such extreme tolerance, even after injury, would be extremely unlikely by denizens of the western world, where conflicts of similar kind often end up in court after retaliatory nest removals or illegal killings^7,75^ and where urban people are often reported as disconnected from nature and profoundly puzzled by conflicts with wildlife, frequently seen as a nuisance to remove^10,18,76,77^. Finally, to date the few studies that have examined the role of human religion, ethnicity, or social factors in human-wildlife conflict have shown how they can shape human attitudes and perceptions and thus intervene on the human side of the conflict^5,7,42,61,73,74^. In our case, we show that they can also affect the animal side of the conflict-interaction by shaping animal aggression through reward and habituation, thus confirming and extending their importance.

### Implications for ethnozoology and the importance of human cultural factors

Human socio-cultural factors permeated all results and allowed more realistic insights into the drivers of a human-wildlife conflict. In particular, kite attacks on humans responded to a geography of human religion, hygiene and poverty, and were concentrated within the productive sector of the kite population located at the high-end of the human-exploitation axis. Notably, kite behaviour was keenly adjusted to humans, tolerating them at close range when feeding but attacking them when provoked, while humans equally responded to kite behaviour, encouraging their ecosystem service function and avoiding them without retaliation when attacked. In this sense, kites and humans could be contextualized as participants in a “coupled-system” where each of the two actors co-shaped each other’s socio-ecological space through repeated interactions, a phenomenon already suggested for other species^78–81^. Human culture was thus key to identifying drivers of attacks and problematic sectors of the city. It also intervened to alleviate the conflict, as current evidence suggested that, at present, the aesthetic, cultural, spiritual and ecosystem-service benefits offered by kites clearly outweighed the local, albeit diffuse discomfort provided by aggressive individuals. This highlights a growing appreciation of the value of intangible benefits provided by wildlife to humans^12,74,82,83^, but most of all, it shows how ethnozoological approaches can improve ecological insight and bridge the gap between different disciplines such as behavioural ecology, wildlife management and urban ecology through direct incorporation of human socio-cultural aspects^2,3^. In fact, human-wildlife conflicts have been identified as prime examples of research and management activities where incorporation of socio-cultural tools is direly needed^3,4^. In conclusion, given that many predatory vertebrates are likely to be attracted by subsidies from a growing human population worldwide^84^, conflicts promoted by close exposure to humans, as portrayed here, are likely to increase.

## Methods

### Ethics statement

This research is part of a larger and long-term study on the demography of Black Kites in Delhi. We received the permits to conduct the fieldwork from the office of the Additional Principal Chief Conservator of Forests (APCCF), the Government of the National Capital Territory of Delhi under the provisions of the Wildlife Protection Act, 1972 (permit number: CF/LC/105/07/HQ/10504-8). The Training, Research, and Academic Council (TRAC) of the Wildlife Institute of India, Dehradun (WII), gave bioethical approval for the research protocols. We performed all methods in accordance with the relevant guidelines and regulations laid out by TRAC WII with respect to study animal and human participants. We also sought informed consent from all the participants (or their legal guardians) for the semi-structured interviews (see below). We took all precautions to ensure researcher and animal safety, and maintained anonymity of the human respondents at all the stages of data recording during the field trials. All members of the field team were regularly administered with preventive vaccination, they wore thick hats/helmets and appropriate protective clothing so as to ensure safety.

### Study Area

Delhi is a megacity of more than 16 million inhabitants, covering an area of 1500 km^2^ and in constant expansion^85^. Three aspects of Delhi are important for kites. First, much of the city is characterized by poor solid waste management, which affords plenty of food to kites in the form of carrion or refuse. Second, many people engage in the centuries-old religious practice of feeding meat scraps to kites (hereafter termed ‘ritualized-feeding’), typically offered by throwing meat into the air for the birds to catch. These offerings are made for a variety of reasons, such as asking for blessings and relief from sins and worries^51,52^. Whilst meat-offering is practiced by a number of communities, in Delhi it is especially prevalent amongst members of Islamic faith, whose numbers are concentrated in well-defined portions of the city (hereafter ‘Muslim colonies’) where large quantities of meat are tossed to kites at predictable hours each day, sometimes causing hundreds of kites to congregate. Third, Delhi retains reasonable green cover, thus providing abundant nesting habitat for kites^86^.

### Fieldwork procedures and statistical analysis

Data on attacks were collected during nest-checks in 2013–2016, conducted at 20 plots randomly scattered throughout the city in order to cover all its possible urban settings, from semi-natural to extremely built-up sites (see^44,50^ for details of plots and nest checks). On each occasion, nests were visited in a standardised manner: a team of three people approached the nest directly from a point approximately 50 m from the nest, chosen to be clearly visible to a kite perched in the nest area. One person (always the same one) then proceeded to climb the nest. A kite pair was classified as attacking when either of the two parents dive-bombed and made physical contact with any member of the team. To examine the characteristics that may affect the likelihood of aggression, we compared attacking and non-attacking pairs in the following manner. First, for each pair that attacked us, we chose a non-attacking pair that: (1) had eggs or chicks of similar age, (2) that was checked in the same year and on the same or preceding-following day, (3) that had received a similar number of previous visits by our team, and (4) that had a similar tree-arrangement configuration (nest in an isolated tree, line of trees, parkland or continuous woodland). This allowed us to investigate aggression while removing the potentially confounding effects of year, date, breeding stage, previous visit and local habitat-structure. Second, for all attacking and non-attacking pairs we collected a number of landscape and human variables (Supplementary Table S.1), based on our knowledge of kite ecology and of a previous study on habitat preferences by Delhi kites^50^. These variables estimated the structure and composition of the urban landscape around the nests, their local availability of organic garbage, their access to Muslim ritual-subsidies, the local density of humans around the nest and in the surrounding streets, and the close exposure to human presence through the presence-absence of an open balcony within 20 m of the nest (details in Supplementary Table S.1). Thus, they characterized each pair on the basis of its surrounding urban characteristics, food availability, and exposure to humans as well as their subsidies. Third, we used a logistic mixed model^87^, with pair-identity nested within plot-identity, to discriminate between attacking and non-attacking pairs on the basis of the landscape and human variables. To reduce collinearity and the number of variables presented to multivariate models, we employed the method of variable reduction proposed by Green^88^. In this method, pairs of strongly intercorrelated variables (r > 0.6) are considered as estimates of a single underlying factor. Only one of the two is retained for analysis, usually the one likely to be perceived as more important by the study organism. Of the remaining variables, only those for which significant univariate differences (P < 0.1) were detected between attacking and non-attacking pairs were included in the logistic model (Supplementary Table A.2). Univariate differences were carried out by means of t-tests and χ^2^ tests. Model building was implemented through an information-theoretic approach, following recommendations by^89–91^. We used the “dredge” function of the MuMIn package to rank competing models on the basis of their weight and AICc^89^. Models within 3 AICc units of the top model were selected for model averaging, implemented through the MuMIn package. All the analyses were performed through R 3.4.3^92^.

Finally, to gain an understanding of the extent and impact of attacks on local communities, we approached and interviewed all the people we encountered during our trials in the immediate proximity of the nests of attacking and non-attacking pairs (N = 278 interviews). This allowed us to test whether pairs that attacked us also had a higher probability of previously attacking local people, i.e. before and independently of our activities. Detailed analysis of the interviews will be reported elsewhere, but in the Discussion we delineate the main local opinions qualitatively, in order to place the conflict in the context of local attitudes. Throughout, all tests are two-tailed, statistical significance was set at ≤0.05 and means are given with 1 SE. The dataset of the current study is available on reasonable request from the corresponding author.

## Supplementary information

Human-attacks by an urban raptor are tied to human subsidies and religious practices Nishant Kumar, Yadvendradev V. Jhala, Qamar Qureshi, Andrew G. Gosler & Fabrizio Sergio

## Data Availability

Given that a part of the data is funded by a Foundation who has shared possession of the generated datasets, the data for the manuscript are available upon reasonable request from the authors.
