# Supplementary material for: Human-attacks by an urban raptor are tied to human subsidies and religious practices
Source: Sci Rep. 2019 Feb 22;9:2545. doi: 10.1038/s41598-019-38662-z (PMC6385285; doi:10.1038/s41598-019-38662-z)
Supplement: Supplementary file 1 — Human-attacks by an urban raptor are tied to human subsidies and religious practices Nishant Kumar, Yadvendradev V. Jhala, Qamar Qureshi, Andrew G. Gosler & Fabrizio Sergio [file 41598_2019_38662_MOESM1_ESM.pdf]

Human-attacks by an urban raptor are tied to human subsidies and religious practices

Nishant Kumar, Yadvendradev V. Jhala, Qamar Qureshi, Andrew G. Gosler & Fabrizio Sergio

Supplementary Tables

Supplementary Table S.1. Ecological, landscape and human variables used to characterize Black kite attacking and non-attacking pairs within the city of Delhi (India).

Variable	Description
Number of people	Number of people (not belonging to the field-team) within 50 m of the nest at the beginning of the attack trial (e.g. sitting in a park, playing, walking in the street, etc.)
Number of offspring	Number of eggs or chicks in the nest at the time of the kite attack trial
Breeding Success	Categorical variable: 0 = failed breeding attempt, 1 = successful breeding attempt. An attempt was classed as successful if the pair managed to raise at least one nestling to fledging age (40-48 days old).
NND5 (m)	Mean of the distance to the five closest kite neighbours
Territories within 200 m	Number of territories occupied within 200 m of the target nest
Colony size	Number of nests within the kite colony
Balcony	Categorical variable: 0 = absence, 1 = presence of a balcony within 20 m of the sampled nest
Index of road density	Number of asphalted roads crossed by a 500 m north-south and a 500 m east-west transect crossing each other on the nest
Urban cover	Percentage area covered by built-up structures (buildings, roads, parking lots, or any other impervious surface) within 500 m of the nest
Green cover	Percentage area covered by shrub/tree vegetation within 500 m of the nest
Hygiene score	Level of sanitation: 1 = clean areas; 2 = areas under poor waste management regime [a]
Human density	Average number of people walking within 2m of a stationary observer during 5 min at 10 locations randomly plotted within 200 m of the nest [b]
Access to Muslim subsidies	First component (PC1) of a principal component analysis on Muslim density and on the proximity to the three closest Muslim colonies (following Kumar et al.[39]).

[a] Categorical variable with two levels: 1 = efficient waste disposal with very scarce or no organic refuse in the streets; 2 = abundant and widespread refuse in the streets throughout the area, either in small frequent piles, in illegal ephemeral dumps, or as individual items scattered a bit of everywhere through all streets (following Kumar et al.[50]).

[b] Counts were only operated between 10:00-17:00 hrs and avoided during atypical, momentary peak periods of human traffic, such as exits from work or schools, in order to maintain consistency across sites (following Kumar et al.[50]).

Supplementary Table S.2. Mean (± 1 SE) estimates of variables measured at 36 Black Kite pairs that attacked humans for nest defense and at 36 control pairs for which no attacks were observed. Differences between the two samples were tested by means of t-tests, or χ^2 tests for categorical variables. Symbols: * P < 0.05; ** P < 0.01; *** P < 0.001.

Variable	Attacking pairs	Non-attacking pairs
Offspring number ***	1.89 ± 0.10	1.32 ± 0.10
Breeding Success [a, b] ***	85.29 %	42.15 %
Index of road density [b]	7.52 ± 0.24	7.40 ± 0.34
Urban cover [b] ***	0.46 ± 0.02	0.35 ± 0.02
Green cover [b] **	0.23 ± 0.01	0.31 ± 0.02
Balcony [b, c] ***	61.76 %	24.51 %
NND5 (m) *	156.31 ± 10.75	197.58 ± 16.66
Territories within 200 m	9.89 ± 0.61	8.85 ± 0.58
Colony size	5.67 ± 0.34	4.92 ± 0.31
Number of people [b] ***	18.05 ± 1.47	8.96 ± 0.83
Hygiene score [b, d] ***	87.25 %	54.90 %
Human density [b] ***	17.38 ± 1.19	11.49 ± 0.72
Access to Muslim subsidies [b] **	0.39 ± 0.11	-0.19 ± 0.07

[a] Percentage of nests which raised at least one nestling to fledging age (40-48 days old).
[b] Variable fitted to the multivariate models of Table 1.
[c] Percentage of nests which had a balcony within a 20 m radius.
[d] Percentage of locations with poor sanitation.
